# Prenatal Neuropathologies in Autism Spectrum Disorder and Intellectual Disability: The Gestation of a Comprehensive Zebrafish Model

**DOI:** 10.3390/jdb6040029

**Published:** 2018-11-30

**Authors:** Robert A. Kozol

**Affiliations:** Department of Biology, University of Miami, Coral Gables, FL 33315, USA; r.kozol1@miami.edu; Tel.: +1-956-284-3973

**Keywords:** autism spectrum disorders, ASD, intellectual disability, ID, zebrafish, prenatal, embryonic, fetal, neurodevelopmental disorders, critical periods

## Abstract

Autism spectrum disorder (ASD) and intellectual disability (ID) are neurodevelopmental disorders with overlapping diagnostic behaviors and risk factors. These include embryonic exposure to teratogens and mutations in genes that have important functions prenatally. Animal models, including rodents and zebrafish, have been essential in delineating mechanisms of neuropathology and identifying developmental critical periods, when those mechanisms are most sensitive to disruption. This review focuses on how the developmentally accessible zebrafish is contributing to our understanding of prenatal pathologies that set the stage for later ASD-ID behavioral deficits. We discuss the known factors that contribute prenatally to ASD-ID and the recent use of zebrafish to model deficits in brain morphogenesis and circuit development. We conclude by suggesting that a future challenge in zebrafish ASD-ID modeling will be to bridge prenatal anatomical and physiological pathologies to behavioral deficits later in life.

## 1. Introduction

Autism spectrum disorder (ASD) and intellectual disability (ID) share diagnostic features, such as deficits in social behavior, and problems with communication and stereotypies [1,2]. As such, individuals with ASD are commonly diagnosed with ID (50–70%) and vis-versa (28–70%) [1,3,4]. These common co-diagnoses are likely a product of environmental and genetic factors that converge on similar molecular pathways [1,5]. Due to the relative diagnostic age (Median ASD diagnosis = 4.5 years; ID = 1–18 years) and enrichment of synaptic gene mutations ASD-ID, animal models initially focused on postnatal time periods [6,7,8,9]. However, studies have recently shown that disruptions in fetal development can manifest as childhood behavioral deficits [10,11]. These studies include zebrafish models that are being used to pinpoint critical periods and to provide a more complete picture of the mechanisms that underlie symptoms [12,13]. 

Zebrafish are increasingly used as a prenatal animal model for neurodevelopmental disorders, due to several anatomical and behavioral characteristics. First, zebrafish are small and transparent during early life stages which facilitates visualization of the nervous system, both anatomically and physiologically [14,15,16,17]. Therefore, developmental disruptions that might impact circuits can be investigated morphologically using transgenics to label specific neuronal populations [18,19], or physiologically using genetically encoded calcium indicators to assay neuronal activity [20,21,22]. Stereotyped swimming behaviors also emerge embryonically and rapidly transition to evasive and predatory behavior [23,24]. Disruptions in critical periods of development can therefore be identified by analyzing these behavioral milestones, such as embryonic tail coiling [25] and larval swimming [26]. Finally, one week old larval zebrafish have a small brain that simplifies studying how circuit-level disruptions affect behaviors at the whole-brain level [22]. Together these attributes produce a cellular- to circuit-level model for establishing functional relationships between prenatal developmental deficits and behavioral phenotypes. 

In this paper, we look at how zebrafish are used to study mechanisms by which causal genetic mutations for ASD-ID impact embryonic development. We review recent studies of prenatal factors affecting ASD-ID, that detail critical time periods and the cellular processes responsible for later behavioral deficits. Then, we look at zebrafish proof-of-concept ASD-ID models that investigated embryonic and larval phenotypes related to ASD-ID. Finally, we conclude by discussing how recent advances in methodology are now being employed to investigate how developmental changes in zebrafish ASD-ID models contribute to behavioral phenotypes. 

## 2. What Prenatal Factors Contribute to ASD-ID

The relationship between prenatal development and symptoms of ASD-ID was first established by investigating maternal teratogen exposure and gestationally derived deficits in ASD-ID syndromes. Teratogens are agents, chemical or infectious, that cause malformations to an embryo or fetus [27]. Many teratogenic effects depend on the time of exposure and can be traced back via hospital records or estimated by linking developmental milestones to somatic defects [27,28]. In addition to teratogens, many genes are expressed prenatally and several well-studied ASD-ID syndromes, such as Tuberous Sclerosis, are known to include gestationally derived malformations [10]. Recent research has expanded these prenatal factors to include maternal cytokines, genetic networks and non-coding genetic contributions [29,30,31,32]. However many individuals with ASD-ID have a non-etiological diagnosis, with neuropathological roots that may stem from prenatal time periods. In fact, some anatomical features arising from prenatal deficits have been described in adults with ASD-ID, including ectopic superior olivary nucleus neurons and loss of cerebellar Purkinje Cells with intact inferior olivary nuclei [33,34,35,36]. These features highlight the need for prenatal animal models to determine whether environmental and genetic risk factors for ASD-ID influence later behavioral deficits.

### 2.1. Environmental Risk Factors

Prenatal critical periods for ASD-ID were initially identified by unintentional population scale exposures to teratogens, that were initially prescribed as medications during pregnancy [37,38,39]. These studies showed that children exposed to teratogens in utero, such as thalidomide or valproic acid (VPA), developed symptoms of ASD-ID later in life [27]. To narrow down critical periods, researchers matched somatic defects with an accompanying developmental window, such as neural tube defects with neural tube closure [27]. For example, some children of mothers exposed to thalidomide developed autistic symptoms and cranial nerve defects consistent with exposure around 20 to 24 days post conception [27,28]. Despite these temporal signatures exposure can occur throughout the entire pregnancy, making it difficult to ascribe prenatal disruptions to later behavioral deficits. To identify whether these critical periods affect post-natal behavior, animal models have been screened for behavioral deficits, following exposure to teratogens at different time intervals. For example, following exposure to VPA during embryogenesis (E10–E13), rats exhibited learning and social deficits as adults [40,41]. Several zebrafish labs have also modeled the teratogenic effects of thalidomide [42] and VPA [43]. A recent study exposed zebrafish to VPA from gastrulation to neural tube formation, mimicking the window of exposure used in mouse VPA models [44]. VPA exposed larval fish showed decreases in histaminergic, noradrenergic and dopaminergic systems; accompanied by changes to larval sensory behavior and adult social behavior [44]. These models highlight the usefulness of testing teratogenic hypotheses from clinical data, to determine critical embryonic time periods that underlie later ASD-ID symptoms.

Another environmental factor that can increase the risk of ASD-ID are maternal immune responses [32,37,45]. In the 1970’s, Chess and colleagues found that mothers exposed to rubella during pregnancy had children with a high incidence of ASD-ID [37]. Recent studies have identified several immune responses, such as higher concentrations of intergerons in ASD-ID versus the general population, or ASD alone [32,45]. Additionally, researchers have shown that inflammatory molecules influence fetal gene expression, including dysregulation of ASD-ID genes associated with mTOR and EIF4E dependent signaling [30]. A recent ASD-ID zebrafish model looked at larval immunological function to determine whether immune responses were affected in a *Methyl-CpG-Binding Protein 2* (*MECP2*) null background [46]. MECP2 mutations cause Rett Syndrome, which includes symptoms associated with inflammation, such as gastrointestinal disturbances and oxidative stress [47]. Mecp2 mutants exhibited decreased expression of *tumor necrosis factor a* (*tnfa)* during early embryogenesis and increased expression of *interleukin-1b* and *interleukin-10* throughout larval stages [46]. Inflammatory molecules from these genes are needed to stimulate the bodies response to a disturbance and promote tissue repair [46]. Therefore, these changes in gene expression may be a response to tissue disturbances or oxidative stress, including a dysregulated inflammatory response of the gastrointestinal tract. These studies provide evidence for an interaction between the environment, inflammation and genetic risk factors in ASD-ID.

### 2.2. Genetic Risk Factors

Syndromes associated with ASD-ID, including Rett, Fragile X Syndrome (FXS) and Tuberous Sclerosis (TSC) identified the first genetic mutations of large-effect to cause ASD-ID [48]. Causal mutations for all of these conditions occur in genes normally expressed during prenatal periods. The *Fragile X Mental Retardation* (*FMRP*) gene is expressed throughout development and produces many alternatively spliced variants. Because different protein isoforms are expressed at different times and in different cell types [49,50], phenotypes vary depending on the nature and position of *FMRP* mutations. For example, while deletion of *FMRP* in humans and mice is linked to cortical defects, a common *FMRP* repeat mutation in FXS does not cause obvious gross anatomical deficits [50]. None-the-less, pluripotent stem-cells derived from individuals with FXS exhibit developmental delay in neuronal differentiation during embryogenesis [51]. Likewise TSC, caused by mutations in *TSC1/*2, is a ASD-ID syndrome and characterized by large benign cortical tubers and giant cells [1,7]. Individuals with TSC have been shown to have gross anatomical defects during mid-fetal development, including focal lesions [52,53] and giant cells [54]. Finally, recent ASD-ID mutational models have functionally linked deficits in prenatal cellular processes, such as neurogenesis, with postnatal ASD-ID behavioral deficits [11,55,56]. These embryonically expressed single gene mutations point to prenatal time periods as being critical to symptom development in ASD-ID.

In addition to these syndromic causes of ASD-ID, the vast majority of individuals have non-syndromic rare genetic variants, or are diagnosed with idiopathic ASD-ID. Fortunately, more high-confidence ASD-ID genes are being identified, as a result of world-wide consortia, and whole-genome and whole-exome sequencing [57,58,59,60]. These studies have shown that ASD-ID gene mutations are heterogenous and rare, implicating hundreds of ASD-ID associated genes. This recent increase in gene discovery, along with transcriptomic and proteomic analysis, has enriched our understanding of molecular networks associated with ASD-ID (recently reviewed in References [9,61,62]. The focus has thus shifted from defining genetic causes of ASD-ID to understanding how biologically relevant networks contribute to ASD-ID symptoms [63,64,65]. These biologically relevant networks are constructed by analyzing gene expression profiles, including gene ontology terms and protein-protein interactions [31,66,67]. These integrated models continue to identify prenatal ASD-ID clusters with greater temporal and spatial specificity, highlighting commonly impacted structures, like the fetal amygdala and mid-fetal cortical projection neurons [31] and molecular pathways, such as Notch and Wnt signaling [66]. In addition to rare genetic variants, common genetic variation of small-effect size also contribute to the risk and development of ASD-ID [61,68]. Although a single variant confers a small increase in ASD-ID risk, cumulative variation across the genome could account for large contributions (15–50%) towards the genetic etiology of ASD-ID [68]. These common genetic variants may also impact the penetrance of rare genetic variants, influencing the development and severity of ASD-ID phenotypes [69]. The ASD-ID genetic landscape continues to expand, with genetic variants and biological networks explaining a large percentage of the ASD-ID etiology.

Maternal genetics can also contribute to symptoms of ASD-ID via maternal effects. Maternal effects are maternally derived material that can contribute to the development of an offspring [70,71]. Maternal effects include embryonic translation of maternally loaded mRNA transcripts, genetic imprinting and the influence of intrauterine factors [70]. While maternally derived mechanisms including 15q duplications and imprinting in Angelman syndrome are well known, recent studies have discovered novel maternal effects. For example, a recent genome-wide association study linked a parent-of-origin effect with the transfer of a maternal ASD-ID gene *SH3 and multiple ankyrin repeat domain protein 3* (*SHANK3*) variant, suggesting that this parent-of-origin variant increased the likelihood of developing ASD [72]. Finally, maternally loaded genetic variants can also affect developing oocytes. Following long-term storage a Drosophila FXS model exhibited dysregulated neuronal development, due to translational deficiencies of transcripts encoding large proteins [73]. This finding supports previous research that identified deficits in translation of transcripts encoding large proteins as a mechanism plaquing local translation at synapses [73,74]. These studies indicate that mutations in genes linked to ASD-ID can influence neuropathology even before the embryo begins to develop.

## 3. Comparative Neurodevelopment and Critical Periods: Can Embryonic and Larval Periods in Zebrafish be Compared to Prenatal Development in Humans?

The use of animal models to study human neurodevelopmental disorders requires the ability to translate critical periods across species. However, this can be problematic due to varying rates of development and dramatically different life histories. For instance, the human prenatal period is defined by prolonged in utero gestation and infants are born dependent on parental care [75,76]. In contrast, zebrafish develop externally in a protective chorion and hatch after a few days as fully independent larvae [14]. Moreover, morphogenesis of the zebrafish brain is complete in three days, compared to twenty one days in mice and eight weeks in humans [14,77]. Therefore, internal neurodevelopmental milestones should be used to define critical periods, from the completion of embryogenesis to the maturation of neural circuits essential for complex behaviors. 

Currently, a comprehensive guide for comparing zebrafish and mammalian neuronal development is lacking. In mammals a group recently made a resource for comparing critical periods, by using internal and external developmental events. These events included the onset and offset of neurogenesis, cellular processes like myelination and the appearance of behaviors [76,78]. This comparative mammalian study revealed that the order of these events is largely conserved across species, despite differences in gestational time and alternative life histories (e.g., precocial versus altricial development). This order of events may also be conserved in zebrafish; however, a major knowledge gap exists concerning periods of extensive brain growth, between late larval and juvenile stages. Fortunately, research in zebrafish embryos have confirmed that major brain regions, gene expression patterns and cell types remain homologous with mammals (Table 1) [79,80,81]. This section focuses on comparing zebrafish and mammalian neural development and a strategy for translating prenatal regional or circuit specific critical periods. 

### 3.1. Conserved Neuronal Development with Differences in Morphogenesis

Although neuronal development is largely conserved among zebrafish and mammals, several differences exist in morphogenesis and neurogenesis [14,81,96]. Zebrafish and mammals utilize different strategies during the formation of the neural tube: while zebrafish develop a neural thickening or keel that is hollowed by cavitation, mammals create a neural tube by enfolding the neural epithelial sheet [14,97]. Although these mechanisms differ, the underlying genes and signaling pathways responsible are largely conserved [97,98]. Morphogenesis of the telencephalon also differs: the mammalian neural tube evaginates and ventricles develop internally (medial), while the zebrafish telencephalon everts and ventricles develop on the surface of the brain [99,100]. This eversion process rearranges the topographic layout of functionally homologous brain regions, that results in a dorsal-medial amygdala and lateral hippocampus. Furthermore, there is still debate in the fish community over the severity of eversion and the final placement of regions within the forebrain [101,102]. Despite morphogenic differences, regions similar to the amygdala [103,104] and hippocampus [105,106] have been identified by conserved gene expression and function [102]. This provides a rationale for using zebrafish to study neuropsychiatric disorders in relation to emotive, social and memory-based behaviors [107,108].

### 3.2. Integrated Critical Periods: Gene Expression, Morphogenesis and Function

Currently zebrafish researchers must utilize molecular markers, cellular processes and circuit function to identify translatable critical periods. Some brain regions such as the cerebellum maintain remarkable genetic, cytoarchitectural and functional homology [80,109,110,111]. As an example of regional developmental staging, milestones of cerebellar development can be used to translate developmentally similar critical periods between zebrafish and mammals (Table 2). First the primordial cerebellum is defined by the expression of *engrailed1/2* in rhombomere 1 of zebrafish (1–2 dpf), mouse (10–12 dpf) and humans (28 dpf) [109,112]. Cerebellar progenitor pools are then specified and can be identified by their expression of the transcription factors *Math1/Atoh1a* and *Ptf1a* [109]*. Math1/Atoh1a* labels the upper rhombic lip that will give rise to excitatory granule cells, while *Ptf1a* labels the ventral cerebellar proliferation zone that gives rise to inhibitory purkinje cells [113,114]. Following neuronal proliferation, the granule, purkinje and deep cerebellar neurons (eurydendroid cells in fish) migrate to assemble a trilaminar structure [113,114]. Critical periods can also be identified as cerebellar regions become compartmentalized for specific functions, similarly to mammals [115,116]. For example, the caudal purkinje cell layer in zebrafish has conserved efferent projections and functions with the dorsal cerebellar vermis in mammals [111,117]. The caudal vermis has indeed been implicated in ASD-ID, including loss of purkinje cells and deficits in saccade adaptation [35,118]. This specific example shows that several criteria can be used to define regional critical periods, including the appearance of a structure, neurogenesis of specific cell types, formation of a functional circuit and emergence of function related to behavior (Table 2). 

## 4. Zebrafish ASD-ID Models: Critical Periods and Developmental Mechanisms

**Figure 1:**
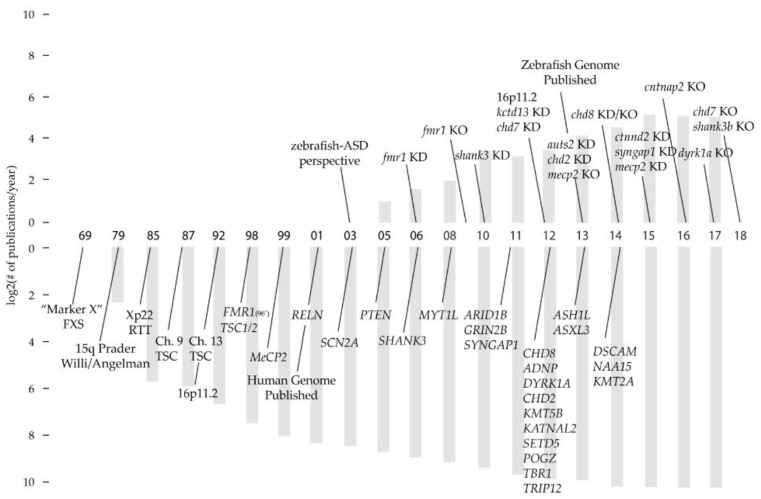
A timeline of ASD-ID genetic discoveries and ASD-ID modeling in zebrafish. Names represent the characterization of a gene associated with ASD-ID or the first report of a genetic variant associated with ASD-ID (bottom). ASD-ID genes where chosen from the Simons Autism Research Initiative (tier 1 genes) and common ASD-ID syndromes. Zebrafish genetic models denote the first reported embryonic or larval model and the method of genetic manipulation, KD = morpholino knock-down, KO = mutant allele. Histograms describe publications per year for PubMed keywords ASD, ID, Genes, Genetics, Loci, Chromosome (top zebrafish, bottom human). Non-linear intervals of years were averaged. Human timeline references: “Marker X” [138]; 15q Prader Wili/Angelman [139]; Xp22 RTT [140]; TSC Ch. 9 [141], Ch. 16 [142]; *FMR1* [143]; *TSC1/2* [144,145]; *MeCP2* [47]; *RELN* [146]; *SCN2A* [147]; PTEN [148]; *SHANK3* [149]; *MYT1L* [150]; *ARID1B* [151]; *GRIN2B* [152]; *CHD8*, *ADNP*, *DYRK1A*, *TBR1* [57]; *KMT5B*, *KATNAL2*, *SETD5*, *POGZ* [153,154]; *TRIP12* [155]; *ASH1L* [31]; *ASXL3* [156]; *DSCAM*, *NAA15*, *KMT2A* [157]. Zebrafish timeline references: zebrafish-ASD perspective [121]; *fmr1* KD [132], *shank3* KD [133], 16p11.2 KD [127], *kctd13* KD [134], *chd7* KD, *auts2* KD [158], *chd2* KD [159], *mecp2* KO, *chd8* KD/KO [160], *ctnnd2* KD [161], *syngap1b* KD [126], *mecp2* KD, *cntnap2ab* KO [125], *dyrk1a* KO, *shank3b* KO [162], *chd7* KO.

Recently there have been several proof-of-concept studies that have used zebrafish to model inherited neuropsychological disorders [108,121,122,123,124,125,126,127,128]. The discovery of high-confidence ASD-ID genes was initially slow, due to genome-wide studies with small sample sizes and an ever evolving set of diagnostic criteria (Figure 1) [59,129,130]. Many of the original ASD-ID zebrafish models represent genetic variants that, with high-confidence, were deemed causal for ASD-ID (16p11.2 CNV, *SHANK3*, *FMRP*, *MET*, *TSC1*) [127,128,131,132,133,134]. Additionally, the majority of ASD-ID zebrafish models were made using morpholino knockdown technology, predating the widespread availability of gene editing techniques to generate site specific knockouts. Interpretations of knockdown phenotypes are limited due to off-targeting concerns and the transient nature of knockdown technologies, restricting analyses to early stages [135]. Comparisons between knockdowns and knockouts of the same gene are complicated by off-target effects induced by morpholinos and genetic compensation seen in mutants ([136] discussed in Section 5.2). Given the recent rate of discovery of causal genetic mutations [9,64], future studies should produce a rich understanding of critical developmental time periods and mechanisms. None-the-less, past ASD-ID zebrafish models illustrate the importance of neuronal critical periods and developmental check points; when initiation of gene expression, activity dependent growth and synaptic refinement are required to build behavioral circuits [137]. 

### 4.1. Disruptions in Morphogenesis and Circuit Development of the Embryonic Brain

Early in development the embryo must undergo major morphological changes to specify neural ectoderm, establish the divisions of the nervous system and grow a brain anteriorly. During gastrulation cells of the blastodisc migrate to engulf the yolk, while cells at the leading edge involute to establish the epiblast (ectoderm) and the hypoblast (endo-mesoderm) [163,164,165]. As gastrulation and neurulation progress, molecules such as *noggin* and *chordin* are expressed to establish anterior brain regions [166]. Due to embryonic accessibility, seminal work in zebrafish helped to further elucidate morphogenic mechanisms that lead to the specification and formation of the forebrain, midbrain and hindbrain [167,168,169]. These studies illustrate how transcription factors and signaling molecules are expressed to pattern the neural tube, including feedback loops to restrict brain regions and produce a myriad of neuronal subtypes necessary to build diverse neural circuits [79,81]. For instance, the mid-hindbrain boundary (MHB) is demarcated by midbrain *Orthodenticle Homeobox 2* (*Otx2*) and hindbrain *Gastrulation Brain Homeobox 2* (*Gbx2*) expression. This MHB is further maintained by the mid-hindbrain program consisting of *Fibroblast growth factor 8* (*Fgf8*), *Wingless* (*Wnt1)*, *Engrailed 1/2* (*En1/2*) and *Paired Box* (*Pax*) genes [81]. These genes form a complex network of regional signaling that differentially restrict gene expression and promote the generation of the midbrain optic tectum and the hindbrain cerebellum [81]. Many early studies focused on genetic mutants that exhibited the loss of a brain region, such as *no isthmus (noi)* and *acerebellar (ace)*, that lacked the mid-hindbrain boundary and cerebellum, respectively [170]. These studies exemplify the importance of tightly controlled cellular signaling to establish boundaries and provide lineage restricted differential growth of brain regions.

#### 4.1.1. The Dysregulation of Morphogenic Genes and Conserved Signaling Pathways Influences Brain Size

Some ASD-ID genes are expressed during embryogenesis, when loss-of-function has the potential to affect the spatial and temporal growth of neuronal tissue [12,72,73]. These changes in growth can be caused by dysregulated expression of genes involved in conserved signaling pathways, many of which are responsible for delineating the boundaries of tissues and brain regions. The morphogenic gene *chordin* encodes a BMP antagonist necessary for neurulation, that is instrumental in determining the size of anterior brain regions [171]. Zebrafish ASD-ID gene models have shown disrupted *chordin* expression during gastrulation that greatly influences the size of brain regions. For instance, knocking-down or knocking-out the ASD gene *chromodomain helicase DNA binding protein 8 (chd8)* in zebrafish causes an expanded expression of *chordin* and the forebrain marker *otx2* [160]. These changes in expression increase the tissue that will give rise to the forebrain, that the authors suggest may underlie macrocephaly and increased brain size seen in a subset of individuals carrying *CHD8* mutations [160]. In contrast to *chordin* expansion seen in *chd8* models, knocking-down D*elta Catenin-2* (*CTNND2*), another gene linked to ASD-ID, causes a shortening and widening of *chordin* expression during epiboly and a reduction in presumptive forebrain tissue [172]. Direct interactions between *CHD8* and *CTNND2* with Wnt signaling components may therefore explain changes in expression of *chordin* and other brain region specific markers [173]. For instance, *CHD8* indirectly influences embryonic cortical progenitors through chromatin remodeling, affecting the expression of several Wnt signaling genes [55]. Additionally, inactivation of beta catenin is known to cause *dlx2* upregulation and *dlx2* expression was increased in the prethalamus of *chd8* morphants [160,174]. Furthermore, upregulated *dlx2* expression coincides with an enlarged midbrain that, in *chd8* morphants, is likely due to an earlier increase in neuronal proliferation [175]. Finally, Ctnnd1 also interacts directly with beta-catenin and *ctnnd1* morphants have reduced *axin* expression that may underlie the changes seen in *chordin* expression [57]. These studies demonstrate how gene mutations can affect gastrulation and neurulation through convergent developmental mechanisms, producing diametrically opposite phenotypes.

Zebrafish knockdown and knockout models of *autism susceptibility candidate 2 (auts2*) and *shank3* also suggest ASD-ID genes play essential roles in brain morphogenesis. *Auts2* morphants are microcephalic, have increased apoptosis and a decreased number of neurons in the forebrain [158,176]. Morphant and mutant models of *shank3* are also microcephalic and appear developmentally delayed [126,162]. *Shank3* microcephaly is likely due to increased cell death or decreased neuronal proliferation. Mid-hindbrain apoptosis was increased in *shank3a* morphants, while *shank3b* homozygous mutants exhibited brain-wide decreases in *HuC:RFP* fluorescence [126,162]. Furthermore, olfactory placodal neurons derived from humans with *SHANK3* microdeletions exhibited developmental phenotypes [177]. These young post-mitotic neurons had smaller cell bodies, more extensively branched neurites and motility deficits, that likely impact early morphogenesis [177]. While these studies provide evidence that ASD-ID genes play important roles during brain morphogenesis, more research is needed to identify the molecular mechanisms underlying these early developmental phenotypes.

Zebrafish knockdown and knockout strategies have also been used to model the ASD associated 16p11.2 deletion syndrome. These 16p11.2 models have identified several genes that may contribute to changes in brain size. With 29 core genes affected, a 16p11.2 knockdown screen identified multiple genes that may contribute to brain growth [127]. A follow up study used genetic knockouts to further investigate developmental phenotypes and found that homozygote *fam57ba* mutations contribute specifically to macrocephaly [178]. In contrast, two 16p11.2 models have focused on *potassium channel tetramerization domain containing 13* (*kctd13)*, with morphant and mutant models producing different results [134,179]. A knockdown and overexpression study found that *Kctd13* caused brain morphogenesis phenotypes in a dosage-dependent manner [134]. *Kctd13* morphants exhibited increased proliferation and macrocephaly, while overexpression of *kctd13* mRNA caused increased apoptosis, decreased proliferation and microcephaly [134]. In contrast, a more recent *kctd13* gene deletion model found no phenotypes associated with head size or neuronal proliferation [179]. These researchers suggest that the discordance in knockdown and knockout phenotypes could either be due to off-target effects that enhance phenotypes in morphants or genetic compensation that masks phenotypes in *kctd13* mutants [179]. These studies highlight the challenges in comparing mutant and morphant phenotypes (see Section 5.2).

#### 4.1.2. Deficits in Neurogenesis Negatively Impact Axonogenesis and Circuit Connectivity

During neurogenesis primary neurons extend axons throughout the nervous system that act as a foundation for future circuits. Primary neurons are born within the first 24 h after fertilization, repeat serially and have large cell bodies with long axons [180,181]. Because later-born neurons navigate to their targets along the primary neuron axons, changes in primary neuron axonogenesis can have a significant impact on the function of mature circuits. As these axons grow, synchronized developmental steps establish a delicate balance of circuit components, eventually supporting a flexible repertoire of behaviors. These steps include the differential expression of signaling pathway genes that guide them to correct targets, activity dependent neurite growth and synaptogenesis, and both neuronal and synaptic refinement [137,182,183]. These events are especially important for maintaining balance of excitatory and inhibitory (E/I) inputs, whose homeostasis is necessary to coordinate complex behaviors [184]. Although circuits are resilient, disruptions to E/I balance are common in ASD-ID, underlying comorbid symptoms such as epilepsy and hypotonia [184,185]. 

ASD-ID mutations have been shown to impact axonogenesis, including several zebrafish models investigating embryonic axonogenesis. The X-linked intellectual disability gene *FGF13* was found to play a role in stabilizing microtubules to control proper extension of growth cones [186]. When *FGF13* is mutated, growth cones overextend past their targets, which likely explains defects in *FGF13* null cortical layering. This axonogenic deficit may also underlie learning and memory deficits seen in mice carrying *Fgf13* LOF mutations [186]. Several zebrafish ASD-ID gene models show similar deficits, for example, the *Disrupted In Schizophrenia 1* (*DISC1*) gene is a negative regulator of Wnt signaling that influences cellular migration, axonal transport and axonogenesis [187]. Both mutant and morphant zebrafish *disc1* models exhibit axonogenesis phenotypes, including the absence of supraoptic tracts and deficits in rhombomeric axon development [122,187]. Importantly, these *zdisc1* morphant phenotypes could be rescued by full-length human *DISC1* mRNA but not by mRNA encoding Bipolar/Schizophrenia causing *DISC1* variants [122]. Axon tract phenotypes have also phenocopied anatomical deficits seen in ASD-ID patients. The RAC1 pathway has been implicated as a common molecular pathway affecting individuals with ASD-ID and *RAC1* gene mutations have been implicated in several developmental disorders [188]. Cerebellar hypoplasia is common in individuals with *RAC1* mutations, while over-expression of patient *RAC1* mutations in zebrafish caused disruptions in cerebellar axonogenesis [188]. As mentioned previously, a recent study knocking-down 16p11.2 genes found that 80% of genes tested showed deficits in axonogenesis, including reduced fasciculation, disorganized morphology and supernumerary axon tracts that could be rescued by the corresponding human mRNA [127]. These results suggest that several 16p11.2 CNV genes are necessary for early brain development and contribute to neurological phenotypes. Furthermore, because axon tracts are shaped by environmental cues, disruptions to morphogenesis and axonogenesis are likely mechanistically linked.

Developing circuits can also be impacted by disruptions in the balance of excitatory and inhibitory (E/I) inputs [184]. Evidence for increased or decreased excitatory (glutamatergic) and inhibitory (GABAergic) neurons have been reported in different zebrafish ASD-ID gene models. Recent zebrafish ASD-ID studies have used markers that specify E/I neuronal populations to assess changes in the number and distribution of E/I components. The ASD-ID genes *SHANK3* and *SYNGAP1* encode post-synaptic density proteins, that are essential for maintaining synaptic strength of excitatory glutamatergic neurons via dendritic spine growth and AMPA receptor expression [189,190]. Morphant models of *shank3a* and *syngap1b* exhibited severe behavioral deficits, including seizure-like bouts, unproductive body undulations and decreased sensitivity to touch [126]. *Shank3a* and *syngap1b* morphants showed broad decreases in inhibitory GABAergic neurons, suggesting a shift towards an increased ratio of excitatory neurons [126]. A zebrafish mutant loss-of-function *Contactin Associated Protein 2 (CNTNAP2)* ASD-ID model also quantified E/I balance [125]. In contrast to *SHANK3* and *SYNGAP1*, CNTNAP2 is expressed in inhibitory GABAergic neurons, localizes voltage-gated potassium channels and plays a role in migration of GABAergic neurons [125]. *Cntnap2ab* mutant zebrafish exhibited a decrease in forebrain GABAergic neurons, night-time hyperactivity and increased seizure susceptibility [125]. To determine the possible neurological basis for these behavioral deficits, a drug screen was performed confirming that *cntnap2ab* mutants were sensitive to GABAergic agonists. Surprisingly, NMDA receptor antagonists in wild-type larvae strongly correlated with mutant phenotypes suggesting that *cntnap2ab* mutations also affect behaviorally relevant excitatory components. This combination of systems level neuronal deficits, behavioral phenotypes and drug screening provide a compelling model for future ASD-ID studies.

Future studies investing changes in E/I balance will require the identification of ASD-ID relevant neuronal subtypes in zebrafish. Many ASD-ID genes are expressed in glutamatergic excitatory or inhibitory GABAergic neurons, including *Neurexin* (*NRX*) and SHANK3, or *Sodium Voltage-Gated Channels Alpha Subunit 1* (*SCN1A*) and *CNTNAP2*, respectively [185,191]. Several of these genes have been selectively knocked-out in specific neuronal subtypes to demonstrate that genetic mutations in those neuronal subtypes are sufficient to cause ASD-ID behavioral deficits [185,192]. For example, knocking-out *Scn1a* in forebrain GABAergic neurons recapitulated several Dravet syndrome phenotypes, including seizure susceptibility [192]. These phenotypes are likely due to *Scn1a* loss-of-function in fast-spiking Parvalbumin positive cell types [192]. Zebrafish *scn1lLab* mutants were recently used to discover novel anti-convulsant molecules. This study provided evidence for a GABAergic component, however cellular level and subtype specific neuropathology was not investigated [193]. Identifying similar Parvalbumin positive GABAergic neurons in zebrafish forebrain could provide a better understanding of whether *scn1Lab* mutations affect the same cell types, while determining how those neurons relate to seizure susceptibility and deficits in behavior. Studies looking at peptidergic cell types in the hypothalamus [194,195] and dopaminergic neurons [196,197] have been well characterized by specific molecular markers. Further studies are needed to define other neuronal subtypes, including a thorough molecular census of glutamatergic and GABAergic sub-populations, to further validate zebrafish ASD-ID models.

## 5. Current Limitations for Using Zebrafish to Study Human Disease and Disorder Genes

Although zebrafish provide many advantages for modeling human genetic disorders, the retention of many duplicate genes after a whole genome duplication and the differences in morphant and mutant phenotypes provide challenges for researchers. For example, duplicate genes in zebrafish require researchers to study two genes in parallel, due to the likelihood that subfunctionalization has split ancestral functions between duplicate pairs [198]. In addition due to off targeting and toxicity with morpholinos and genetic compensation in mutants, knockdown and knockout models of the same gene can present different phenotypes [135,199]. Fortunately these issues are not barriers to using zebrafish for disease models and can be tackled via careful consideration while planning projects.

### 5.1. The Teleost Whole Genome Duplication

A whole genome duplication occurred during the evolution of teleosts (zebrafish infraclass) and resulted in the retention of many disorder related genes in zebrafish [200]. When a duplicate is retained, the duplicate pair either share ancestral functions (functional redundancy), split functions (subfunctionalization), or develop new functions (neofunctionalization) [201]. In an effort to resolve the ancestral state of zebrafish genes, recent studies have compared genes in the spotted gar (diverged before the Teleostei duplication) with zebrafish, to determine which gene within a pair is most similar to the ancestral orthologue [198,202]. This method can also be combined with gene expression data (temporal and spatial patterns) and genetic manipulation, to determine whether a gene duplicate is relevant to a human disorder [202]. A recent study showed that a majority of duplicate pairs experience subfunctionalization, requiring double knockouts for disorder modeling [203]. Although only a minority of zebrafish duplicates have been retained (~13%), [203] developmental disorder duplicates have been retained at a much higher rate (~60%) [12]. Therefore, the majority of ASD-ID gene orthologues are likely duplicated and will require studying two genes. Although studying two genes becomes a daunting task, clear advantageous can be garnered from studying a duplicate pair. For example, subfunctionalization could provide a means for teasing out the relationship between phenotypes. One gene may retain a role in ASD-ID social behavior while the duplicate retains a role important for a comorbid phenotype, such as intestinal distress. While subfunctionalization may be advantageous for studying certain genotype-phenotype relationship, gene duplicates remain challenging to study, and researchers need to utilize improved methodology for evaluating ancestral functions when starting a project. 

### 5.2. Gene Knockdowns Versus Knockouts

Genetic knockdown and knockout technologies have strengths and weaknesses when modeling human disorders [199]. Gene knockdown techniques rely on anti-sense morpholinos or short-hairpin RNA molecules, to either inhibit mRNA translation or disrupt pre-mRNA splicing [135]. Gene knockdown technologies have been used extensively to functionally validate human disease genes [204,205,206,207,208]. However, in some cases, morpholinos cause severe phenotypes due to off-target effects, activation of p53 cell death pathways and toxicity [135,209,210]. Alternatively, some morpholinos faithfully phenocopy stable mutant phenotypes that can be rescued by injecting mRNA encoding the targeted gene [170,211,212]. In these cases morpholinos can complement mutant studies, allowing loss-of-function phenotypes to be studied rapidly in multiple transgenic zebrafish lines. Because gene knockdowns are transient, phenotypes must be studied in the embryo [135,213]. Therefore, gene knockdowns could be useful for future embryonic models of ASD-ID and researchers should follow the guidelines for using morpholino knockdown technology. These guidelines detail the proper control experiments needed for morphant studies and suggest using morphants as a complement to genetic mutants [213]. 

More recently, CRISPR-Cas9, TALENS and zinc finger endonucleases have provided a powerful tool, not only to produce gene knockouts but to induce site-specific mutations and knock-ins [214,215]. In some cases, phenotypes are subtler than expected due to genetic compensation, though the pervasiveness of such compensation has yet to be determined [136,216]. None-the-less, targeted knockouts and tailored mutants provide a better representation of the genetic state in human disorders, in comparison to morpholinos [209]. Additionally, when mutagenesis efficiencies are high, phenotypes can be assessed in embryos injected with endonucleases [217,218]. This provides the same expediency and versatility as morpholinos, but without the drawbacks of toxicity and transient phenotypes [217,218]. Therefore when phenotypes are present, knockout models should be used as the primary model for studying genetic causes of human disorders.

## 6. The Integrated Model: Utilizing Emerging Technology to Integrate Anatomy, Physiology and Behavior

These initial ASD-ID developmental models provide windows into the anatomical changes, mechanisms and critical periods that relate to prenatal neuropathology. However, zebrafish neuropsychiatric models can reach their full potential by using an integrative methodological approach. Future studies can bridge the gap between anatomy, physiology and behavior by combining systems level developmental changes (e.g., decreased proliferation), with specificity to neuronal subclasses (e.g., decreased dopaminergic neurons) and behavior (e.g., decreased dopaminergic drive affects motivated behavior). Ideally the next generation of models will utilize innovative methods including, brain atlases providing molecular marker libraries to interrogate anatomical and physiological causes of behavioral phenotypes, and small molecule screens for drug discovery. 

### 6.1. Characteristics of Behaviors during Zebrafish Embryonic and Larval Development

Zebrafish are a powerful prenatal model because precocious embryonic and larval behavior provide an early indicator for disruptions of neural circuits. Prenatal disruptions of neural circuits may go undetected in humans, because ASD-ID diagnostic behaviors emerge slowly during post-natal development. Therefore, a delay between circuit disruption and the emergence of diagnostic behavior might result in prenatal etiological knowledge gaps. These gaps can easily be addressed by studying emerging behavior in zebrafish gene models of ASD-ID. During embryogenesis zebrafish tails begin to bend spontaneously (18 hpf), due to large calcium depolarizations flowing from head to tail [23,219]. Early synchronous behavior then transitions into fully functional swimming by 28 hpf, including touch evoked evasive movements [23]. Importantly, these behaviors utilize conserved midbrain, hindbrain and spinal cord circuits that drive ambulatory movement in humans [220,221]. Zebrafish begin to wire and integrate sensory-motor systems during larval development needed to avoid predators and capture prey [24]. These sensory provoked behaviors have extensive background literature, including functional studies detailing the relationship of sensory input to motor output [22,111,222,223]. This attribute could provide the most compelling translational research from zebrafish ASD-ID models, as sensory based deficits are common in ASD and were recently added to the diagnostic criteria [224,225]. Therefore, we argue that emergent behavioral phenotypes can be used in zebrafish ASD-ID models as a means to assay human prenatal developmental milestones.

Zebrafish also develop social and memory-based behaviors during larval development [24,226]. Social behaviors are important for modeling ASD-ID and several groups have established larval assays, that include shoaling and kin recognition [107,227,228]. However, these behaviors emerge at later stages (>9 DPF) and, although likely conserved with mammals, the neural circuitry remains largely unknown [229]. Studies in adult zebrafish have established assays and provided insight into oxytocinergic and dopaminergic circuits involved in social interactions [230]. Additionally, a novel social interaction paradigm was recently developed that identified forebrain cholinergic neurons essential for social orientating [231]. These emerging behaviors also evoke similar comparative questions discussed in Section 3, such as, what are the neurodevelopmental events and circuits that correspond to these complex behaviors? The zebrafish community will need to address these knowledge gaps in order to interpret circuit-level changes in larval social behavior. 

### 6.2. Brain Atlases and High Resolution Morphometry

Research focusing on structural and functional mapping of the brain has recently made impressive progress. Subtle anatomical changes are common in ASD-ID and will require analyzing microstructure of brain circuits in zebrafish ASD-ID gene models [33,232,233]. Zebrafish may be well suited for studying these subtle anatomical changes due to their smaller brains. Several zebrafish groups have established community brain atlases and libraries using brain registration methods [19,234,235,236,237]. Confocal stacks of larval brains are orientated to a reference brain in a specified *x*, *y*, *z* axis using non-linear registration [238]. These registration methods provide sub-cellular accuracy and ensure that brain regions and neuronal populations overlap properly. Reference brains are then used to map libraries of molecular markers, to delineate neuronal subpopulations and circuits within the brain. Therefore, users can register confocal stacks of neuronal activity (e.g., genetically encoded calcium indicator, GECI) or transgenic backgrounds (e.g., vesicular glutamate transporter 2, *vGluT2a*:DsRed transgenic line [239]), to determine discrete sources of physiological and anatomical phenotypes. Furthermore, some of these analytical methods provide fine structural detail that could be missed analyzing gross anatomical regions by hand. For example, many studies looking at macro- and microcephaly measure changes to the entire brain or conspicuous regions (e.g., between the optic tecta [160]). However, innovations in mapping allow users to analyze specific somas or neuropil in fine detail. A new registration and brain segmentation method was recently used to provide a neuroanatomical map based on transgene expression profiles [235]. This method was able to determine significant differences in microstructure of larval brain regions exposed to VPA [235]. VPA exposed larvae displayed several anatomical deficits, including a decrease in excitatory components of the statoacoustic ganglion. Consistent with this structural alteration, VPA-exposed larvae showed decreased responsiveness following an acoustic startle stimulus. These resources have overcome challenges in analyzing circuits, allowing the community to link anatomical and physiological changes to behavioral phenotypes.

### 6.3. Whole-Brain Visual Outputs of Neuronal Activity

Larval zebrafish have a small brain (~100,000 neuron at 6 dpf) in comparison to adult humans (80,000,000,000), allowing for brain-wide neuronal activity monitoring using a neuronal activity indicator [22]. This includes immediate early genes (IEGs) and genetically encoded calcium indicators (GECIs). Although both are readily available for any lab to use, the former (IEGs) are more accessible to be captured on a typical confocal microscope and analyzed using a community brain atlas. 

IEGs are genes and proteins whose expression increases exponentially following an action potential [240]. IEGs, such as cFOS, are commonly used as a proxy for neuronal firing. However, cFOS expression peaks around an hour following an action potential, making it impossible to use for transient stimulus provoked behavior [241]. In contrast, ERK protein is phosphorylated (pERK) within seconds of neuronal firing and phosphorylation peaks within five minutes [242]. pERK expression can provide information on activity changes following a temporally short stimulus, such as a light flash or vibration. Recently, pERK expression was coupled with a brain mapping technique to provide regional discrimination of changes in neuronal activity [234]. This MAP-Mapping technique takes normalized pERK expression in two groups and computes the difference in expression between comparable anatomical regions [234]. For example, the GABA potentiator pentylenetetrazol (PTZ) causes uninhibited excitatory activity throughout the brain that can be visualized in MAP-Map showing an increase in pERK throughout the brain in comparison to pERK expression with no PTZ. This method provides an unbiased way to survey activity changes in the entire brain during a disorder relevant behavior. 

Calcium indicators become more fluorescent upon binding calcium and are used to study nervous system activity. Calcium indicator dyes have been utilized by zebrafish researchers for decades, because dyes can be injected into blastomeres and backfilled in the spinal cord to label neurons [243,244,245]. This system has advantages over Genetically Encoded Calcium Indicators (GECI), because mutant backgrounds do not have to be outcrossed to GECIs. However, GECI methods have been refined and are now sensitive enough to record whole-brain activity in awake behaving fish [22,246]. Labs have provided a wealth of information on zebrafish brain activity underlying specific behaviors during early larval behavior (6–7 dpf), including response to visual and auditory stimuli [17,20,22,25]. Typically, zebrafish need to be paralyzed or restrained in low melt agarose for recording, however two labs have recently built robotic light-sheet microscopes that can follow freely swimming zebrafish larvae in a dish, while recording GCaMP whole-brain imaging [247,248]. Future studies will be able to follow zebrafish ASD-ID models during complex sensory or social behavior, allowing researchers to define, in real-time, the neural circuit deficits underlying ASD-ID behavioral phenotypes. These methods will be revolutionary in determining whether conserved brain regions are utilized during larval social behavior and memory to model core ASD-ID deficits.

Finally, a new approach called Multi-MAP has combined fixed and live imaging techniques to assess modulations of brain activity by arousal pathways [249]. This approach uses GCaMP6s transgenic fish to record neuronal activity in live fish, followed by fixation and immunostaining to identify changes in different neuromodulatory neuronal sub-classes. A looming stimulus was used to measure reaction time and determine alertness [249]. Fast and slow alertness was found to be correlated with the firing of specific subtypes of neuromodulatory neurons in the locus coeruleus, tegmentum and hypothalamus [249]. Importantly, alertness correlated brain regions showed similar activity patterns in mice, suggesting that the relationship between alertness circuits and behavior are conserved [249]. Furthermore, these conserved circuits and behaviors are important for ASD-ID research because arousal pathways have been implicated in ASD-ID [191,250,251]. This method could be strengthened by functionally testing circuits using lines from a Gal4 or Cre-Lox library [19,252]. The multi-MAP approach leverages the power of live whole-brain activity monitoring and molecularly defined circuits to discover cellular elements underlying behavior. 

### 6.4. Small Molecule Screening and Drug Discovery

Zebrafish larvae are small and take up small molecules through the water column, macking zebrafish attractive for medium-throughput drug screening. Small molecules can be dissolved into water or embryo media, and automated behavioral rigs can test 96 fish simultaneously [253]. Additionally, outbred zebrafish lines provide a heterogeneous animal model that reflects the genetic diversity seen in humans [254]. A potential drug discovery should therefore be attractive for preclinical trials when a phenotype-drug relationship is clearly reproducible. Seminal work in small molecule screens have discovered novel compounds that affect sleep [125,255], the photomotor response [256], seizures [193] and behavioral effects of neuropsychological drugs [257]. A drug screen recently correlated changes in behavior between known neuropsychiatric drugs and novel molecules [257]. By using the drug haloperidol, this method validated the therapeutic efficacy of structurally similar compounds [257]. Identifying alternative molecules to treat neuropsychiatric disorders is important, because many neuropsychiatric drugs target multiple neuromodulatory systems that results in unwanted side-effects. Using this method, zebrafish ASD-ID gene models could be screened for small molecules that recapitulate the therapeutic effect of a marketed drug, while lessoning the effect of unwanted side effects. This strategy could be important for currently prescribed ASD medications, such as risperidone and lithium, that cause severe side effects [258,259]. Finally, whole-brain activity methods could be combined with drug screens to determine relationships between drug influenced circuits and improvements in behavioral deficits.

## 7. Conclusions

Past proof-of-concept studies have established zebrafish as a powerful model for studying prenatal neuropathologies underlying ASD-ID. Prenatal risk factors for developing ASD-ID have been well-documented for over a half century, yet there is still little known about the earliest developmental origins of these disorders. In addition, geneticists are beginning to identify prenatally expressed genes that constitute risk factors for ASD-ID. The current body of zebrafish ASD-ID literature has provided a groundwork for studying broad mechanisms underlying ASD-ID neuropathology, such as brain morphogenesis and circuit development. However, the majority of these studies involved gene knockdowns and future genetic mutants will be needed to determine the link between ASD-ID gene mutations and developmental phenotypes. These mutant studies will also need to integrate the current technological leaps, linking anatomical phenotypes to physiological mechanisms underlying ASD-ID relevant behavior. 

## Figures and Tables

**Table 1:** A timeline of neurogenic events in mammals and zebrafish. Time periods represent approximate dates related to morphological delineation, onset of neurogenesis and onset of behavior. Time periods are approximated as days post-fertilization (dpf).

Structure or Region	Estimated Onset of Development (dpf)		
	Human [75,78]	Rat [75,78]	Zebrafish
Neural tube	21	10	0.4–0.8 [14]
Telencephalon, Diencephalon, Mesencephalon, Rhombencephalon	28–33	11	0.7–0.75 [14]
Cerebellar primordia	28	12	0.8 [14]
Optic tectum	28	12	1 [82]
Hypothalamus, pineal (epiphysis)	28	12	0.75, 1 [83]
Raphe complex	28 [84]	10.5 [85]	3 [86]
Locus coeruleus	28	11	1 [87,88]
Inferior olive	30	11	3 [89]
Medial longitudinal fasciculus	30	11	1.2 [90]
Thalamus	36	14	2 [91]
**Cell type**			
Retinal ganglion cells	33	12	1.2 [92]
Mitral cells	33	12	1 [93]
GABAergic neurons	52	15	1.2 [94]
**Behavior**			
Walking (zebrafish, swimming)	455	48	1.13 [23]
Acoustic startle response	265	29	4 [95]

**Table 2:** Critical periods in the development of the cerebellum. Time periods represent approximate dates related to onset of neurogenesis, function and formation of structure. Time periods are approximated as days post-fertilization (dpf).

Developmental Event	Estimated Onset of Development (dpf)	
	Rat [112]	Zebrafish [109,113,119]
Cerebellar primordia (*En1/2*, *Wnt1*, *Pax2*, *Gbx2*)	12.5	0.8
Progenitor pools specified (*Atoh1a/Ptf1a*)	10.5	1–2
GC/PC migration-differentiation	12.5–15	3
Purkinje cells functionally mature	29.5 [120]	4
Trilaminar structure formed	35–42	5
